# Efficacy and safety of sintilimab in the treatment of sqNSCLC

**DOI:** 10.12669/pjms.42.2.9834

**Published:** 2026-02

**Authors:** Peipei Wang, Yajing Zhang, Guoying Li, Yuehong Hou, Yan Zhang, Cheng Xiang

**Affiliations:** 1 Peipei Wang Department of Oncology, Shijiazhuang People’s Hospital, Shijiazhuang 050000, Hebei, China; 2 Yajing Zhang Department of Oncology, Shijiazhuang People’s Hospital, Shijiazhuang 050000, Hebei, China; 3 Guoying Li Department of Oncology, Shijiazhuang People’s Hospital, Shijiazhuang 050000, Hebei, China; 4 Yuehong Hou Department of Oncology, Shijiazhuang People’s Hospital, Shijiazhuang 050000, Hebei, China; 5 Yan Zhang Department of Oncology, Shijiazhuang People’s Hospital, Shijiazhuang 050000, Hebei, China; 6 Cheng Xiang Department of Oncology, Shijiazhuang People’s Hospital, Shijiazhuang 050000, Hebei, China

**Keywords:** Efficacy, Sintilimab, sqNSCLC, Safety

## Abstract

**Objective:**

To investigate the efficacy and safety of sintilimab in the treatment of sqNSCLC.

**Methodology:**

This retrospective study enrolled 64 patients with advanced sqNSCLC receiving intervention at the Shijiazhuang People’s Hospital according to the inclusion and exclusion criteria between January, 2022 to December, 2024 and were randomly divided into the observation and control groups, each including 32 patients with advanced sqNSCLC. The patients in the observation group received gemcitabine and sintilimab, as well as gemcitabine plus cisplatin, whereas patients in the control group received sintilimab sequentially with gemcitabine plus cisplatin. Compared to each other to evaluate the efficacy and safety of sintilimab in the treatment of advanced sqNSCLC.

**Results:**

After four cycles of treatment, the ORR of the control group patients with advanced sqNSCLC was significantly better than that of the observation group(*P*< 0.05), and no significant difference was found in the DCR(*P*> 0.05); however, the control group’s DCR was slightly higher than that of the observation group. During the treatment course, no deaths due to adverse reactions were recorded. The adverse reactions were mainly distributed in Grades-1 and 2; the incidence of adverse reactions in the control group was significantly lower than that in the observation group (*P* < 0.05).

**Conclusion:**

Sintilimab, as a treatment option for advanced sqNSCLC, can safely improve the effectiveness of intervention in advanced sqNSCLC and prolong the survival of patients with sqNSCLC, with a good safety profile.

## INTRODUCTION

Lung cancer is the leading cause of morbidity and mortality in patients with malignant tumours. sqNSCLC is a common tissue type of lung cancer, accounting for 20%-30% of all NSCLC cases.^1^,^2^ Compared to other types of NSCLC, sqNSCLC originates from squamous epithelial cells in the lungs and is mainly distributed in the central region, which is prone to accumulating large blood vessels and endangering lives.^3^ Currently, treatment for sqNSCLC includes surgical resection, immunotherapy and radiotherapy.^4^ The early symptoms of sqNSCLC are similar to those of respiratory infections, and in most patients, sqNSCLC has already progressed to an advanced stage at the time of diagnosis, which prevents radical surgical treatment. Thus, radiotherapy is the main treatment for sqNSCLC; but the intervention effect of this treatment regimen is relatively poor.

The five-years survival rate of advanced sqNSCLC is 17.8%.^5^ Immune checkpoint therapy is a hotspot in the research and development of tumour therapy. Compared to other interventions, immune checkpoint therapy can inhibit T-cell proliferation and activate blocking signals to enhance the recognition and killing of tumour cells by T cells. Studies have confirmed that immune checkpoint therapy can enhance the efficacy and benefit of first-line treatment regimens for malignant tumours and improve the survival rate of malignant tumour patients.^6^,^7^ Sintilimab, a Chinese self-developed immune checkpoint therapeutic drug, is a recombinant, fully human IgG4-type PD-1 monoclonal antibody that can bind to PD-1 and restore the endogenous anti-tumour T-cell response. Compared to other anti-PD-1 antibodies, sindilizumab has high affinity, significant efficacy, low drug resistance and other characteristics and has unique advantages in malignant tumour treatment.^8^-^10^

Sindilizumab has been approved for marketing by the General Administration of Pharmaceutical Administration of China and is included in the first-line treatment for lymphoma, advanced non-squamous small-cell lung cancer and other diseases. Relevant studies have revealed that the use of sindilizumab as a first-line treatment for NSCLC can enhance the intervention utility of NSCLC and has a high safety profile.^11^,^12^ To confirm the safety and efficacy of sintilimab in sqNSCLC intervention, the present study enrolled patients with advanced sqNSCLC from the Oncology Department of Shijiazhuang People’s Hospital as study participants. The patients were treated with sintilimab based on the radiotherapy and chemotherapy intervention program to explore the effectiveness and safety of sintilimab in advanced sqNSCLC, provide a reference basis for the further development of sqNSCLC treatment and improve the safety of NSCLC.

## METHODOLOGY

This retrospective study recruited 64 patients with advanced sqNSCLC admitted to Shijiazhuang People’s Hospital between January 2022 and December 2024 and divided the patients into observation and control groups according to their treatment protocols. Data were retrieved from the hospital information and management system, collected their various information of all patients.

### Ethical Approval:

The study was approved by the Institutional Ethics Committee of Shijiazhuang People’s Hospital (no. 2024003; Date: February 5, 2024), and written informed consent was obtained from the participants.

### Inclusion criteria:

- Meeting the diagnostic criteria of advanced sqNSCLC.
- Receiving systemic anti-tumour therapy for the first time.
- Having ≥1 measurable lesion.
- Expected survival ≥3 months.
- No brain metastasis with stable symptoms after treatment.
- Obtained the informed consent of the family.

### Exclusion criteria:

- Pathological histology of non-squamous NSCLC and clinical cellular carcinoma component ≤50%.
- History of tumour immune checkpoint inhibition therapy.
- History of solid-organ living blood system transplantation.
- History of central nervous metastasis.
- Need for long-term corticosteroid therapy.
- History of immunodeficiency-type diseases.

### Treatment regimen:

The treatment regimen received by patients in the observation group was gemcitabine plus platinum chemotherapy: gemcitabine hydrochloride 1 g/m^2^ intravenous drip for 30 minutes on days one and eight. Cisplatin 75 mg/m^2^ was injected intravenously for one hour on day one, and four cycles of treatment were conducted for 21 days. Conversely, sintilimab was added to the observation group’s treatment program as follows: gemcitabine hydrochloride 1 g/m^2^ was injected intravenously for 30 minutes on days one and eight. Cisplatin 75 mg/m^2^ was administered intravenously for one hour on day one, and sintilimab 200 mg was administered intravenously on day one. Four cycles of treatment were administered over 21 days. All patients received regular follow-up after treatment, and the follow-up endpoint was progression-free survival (PFS). During the course of chemotherapy, patients received fluid infusion as appropriate and prompt management of adverse reactions. All operations were performed by the same group of doctors.

### Evaluation criteria:

Clinical efficacy, PFS and incidence of adverse reactions were evaluated in the two groups. Clinical efficacy was assessed with reference to RECIST1.1, with baseline tumour assessment before the first treatment, followed by tumour imaging assessment every 30 days and every 45 days after three months. Assessments included complete remission, partial remission, disease progression, stable disease, objective remission rate (ORR) and disease control rate (DCR). Adverse reactions were recorded and assessed using CTCAE 4.03.

### Statistical approach:

Data were statistically analyzed using SPSS26.0, and the measurement data were described using mean ± standard deviation, and the T-test completed the test. Count data were statistically described using percentages, and the chi-square test was utilized to complete the test. *P* < 0.05 was considered statistically significant.

## RESULTS

Overall, 64 patients with advanced sqNSCLC were enrolled between January 2021 and December 2022, with an age range of 48-74 years and a median age of 60, which included a total of 34 male and 30 female patients. Twenty patients had a pathological stage IIIB/C, and 44 had a pathological Stage-IV. The demographic and baseline disease characteristics of the two groups did not show significant differences (*P* > 0.05) (Table-I). Both groups received a four-weeks treatment, and no cases of drug discontinuation were recorded. After the intervention, the ORR of the control group was significantly better than that of the observation group (*P* < 0.05). No significant difference was noted in DCR (*P* > 0.05); however, the DCR of the control group was slightly higher than that of the observation group (Table-II).

**Table-I:** Demographic and baseline characteristics.

Factor		Observation group (n = 32)	Control group (n = 32)	X^2^	P
Age	≤60	7	9	3.59	0.08
	>60	25	23		
Sex	Male	20	14	3.66	0.27
	Female	19	11		
Disease stage	IIIB/C	9	8	0.14	0.59
	IV	23	24		
ECOG PS	Yes	0	0	2.35	0.76
	No	32	32		
Smoking status	Yes	22	21	6.75	0.28
	No	10	11		
Genius loci	Essence	8	7	0.76	0.18
	Liver	11	8		
	Bone	13	17		
Hydrothorax	Yes	10	9	3.51	0.11
	No	22	23		

**Table-II:** Evaluation of therapeutic effects.

Therapeutic efficacy	Observation group (n = 32)	Control group (n = 32)
CR	0	0
PR	8	17
SD	15	10
PD	9	5
ORR	25	53.13
DCR	71.88	84.38

**Fig.1:**
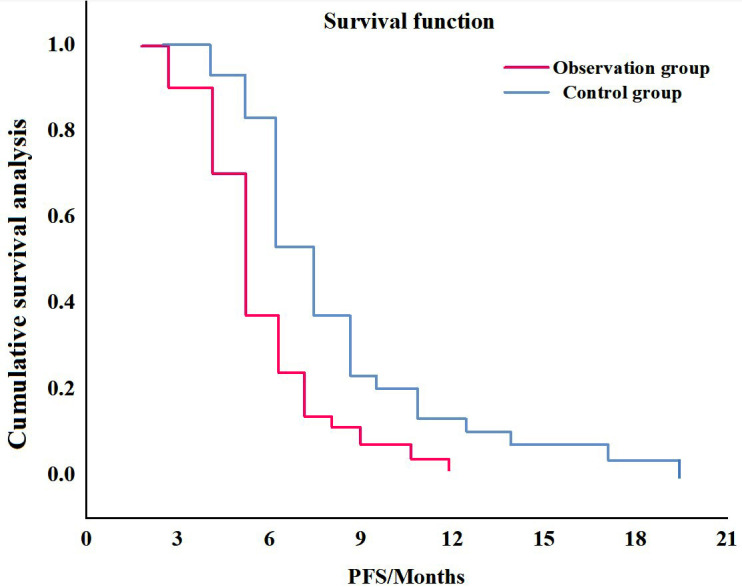
Survival curve.

As of December 2024, the median PFS of the control group was 8.1 months, and that of the observation group patients was 5.8 months; hence, the median PFS of the control group was significantly better than that of the observation group. Fig.1 shows the specific survival curves. No deaths due to adverse reactions were recorded in this study, and the incidence of adverse reactions in the treatment course was anaemia > neutropenia > thrombocytopenia > nausea and vomiting, and the adverse reactions were mainly distributed in grades 1-2. The incidence of adverse reactions in the control group was lower than that in the observation group (*P* < 0.05); Table-III shows the specific adverse reactions.

**Table-III:** Adverse reactions.

Adverse reaction	Observation group (n = 32)		Control group(n = 32)	
	Level 1-2	Level 3-4	Level 1-2	Level 3-4
Anaemia	16	5	14	3
White blood cell count decreased	13	3	12	1
Platelet count decreased	12	3	12	0
Nausea and vomiting	11	1	9	0
Pneumonitis	2	0	1	0
Diarrhoea	5	1	0	0
Erythra	4	0	0	0
Arrhythmia	5	0	0	1
Hypoproteinaemia	7	1	0	0
Hypothyroidism	2	0	0	0

## DISCUSSION

In the present study’, the efficacy and safety of sintilimab in the treatment of sqNSCLC were investigated using a controlled method in patients with advanced sqNSCLC admitted to Shijiazhuang People’s Hospital. According to the results of the study, compared to the traditional platinum-containing double-drug regimen, sequential sintilimab can improve the partial and objective remission rates of patients with advanced sqNSCLC and disease control rate of advanced sqNSCLC to a certain extent. with the results of the ORIENT-12^13^, our results confirm that sintilimab enhances the efficacy of the intervention in advanced sqNSCLC and prolongs the survival time of this patient group. Several studies have confirmed that sintilimab has high tolerability and anti-tumour utility, and its use in solid tumour treatment efforts has a high ORR and safety and can be used as the main option for the treatment of refractory malignant tumour relapse.^14^,^15^ ORIENT-12 explains that, relative to a placebo combined with the platinum-containing dual-drug regimen and the platinum-containing dual-drug regimen in NSCLC, adding sintilimab can effectively enhance the survival of NSCLC patients and improve clinical efficacy and has high safety.^13^ Adverse effects are crucial for evaluating the safety of first-line therapeutic regimens for malignant tumours, and the main adverse effects of sintilimab in combination with platinum-containing dual-agent regimens in the current study were anaemia, neutropenia, thrombocytopenia and nausea and vomiting, which were consistent with the results of the study in ORIENT-3.^16^ Anaemia, neutropenia, thrombocytopenia and nausea and vomiting are expected adverse effects associated with platinum-containing dual-agent regimens, which are almost controllable and can be controlled with targeted interventions. The study results show that, compared to the platinum-containing dual-drug regimen, the overall incidence of adverse reactions during the combination of sintilimab is lower, indicating that the combination of sintilimab and platinum-containing dual-drug regimen has a higher degree of safety.

Currently, lung cancer is the leading cause of cancer deaths worldwide, and NSCLC is the main component of lung cancer, accounting for about 85% of lung cancers.^17^,^18^ According to the site of origin of NSCLC, it can be classified into adenocarcinoma, squamous carcinoma, large cell carcinoma, adenosquamous carcinoma and other types. sqNSCLC is a common histological subtype of NSCLC, accounting for 20%-30% of all lung cancers, and is a malignant tumour with a high incidence of NSCLC. Owing to its unique epidemiological, histopathological and molecular features, the difficulty of sqNSCLC treatment is significantly higher than that of other NSCLCs.

The first-line regimen of platinum-containing two-agent chemotherapy for sqNSCLC has an effective rate of 30%, a PFS of five months and an overall survival of ten months, which is not promising for the clinical efficacy and prognosis of sqNSCLC.^19^ To further enhance sqNSCLC treatment and prolong the survival of patients with sqNSCLC, therapeutic modes and drugs that can enhance the clinical efficacy of sqNSCLC should be searched.

Under the background of the development of medical technology level, immunotherapy has become the main research direction for the treatment of malignant tumours, compared to other intervention programs; immunotherapy can stimulate the mobilisation of the body’s immune system and enhance the anti-tumour immunity to achieve the purpose of killing tumour cells and controlling the progression of the tumour disease. Sintilimab is an independently developed immunotherapeutic drug in China released in 2018 and belongs to the PD-1 class of monoclonal antibody drugs. The drug was approved for marketing in China in December 2018 and was included in the clinical treatment program for lymphoma in 2019 and the first-line treatment program for non-squamous NSCLC in 2021.^20^

### Limitations:

Although the present study confirmed the efficacy and safety of sintilimab in sqNSCLC treatment, owing to the limitation of time and manpower, the present study had a limited sample size and insufficient follow-up time, which did not allow for the comprehensive analysis of the biomarkers related to sqNSCLC and evaluation of the OS benefit. In subsequent studies, a larger sample size, longer follow-up time and a more comprehensive study are warranted to explore the advantages of sintilimab application.

## CONCLUSIONS

The combination of sintilimab and platinum-containing dual-drug regimen in the treatment of advanced sqNSCLC can improve the ORR and prolong the survival of sqNSCLC patients, with better safety. Further studies on the application of this regimen in subsequent interventions are required.

### Authors’ Contributions:

**YZ** and **PW:** Carried out the studies, participated in collecting data, and drafted the manuscript, and are responsible and accountable for the accuracy or integrity of the work.

**GL, YZ, CX** and **YH:** Performed the statistical analysis and participated in its design.

All authors read and approved the final manuscript.
